# Pitfalls in the management of subglottic paragangliomas at unusual location: a case report and literature review

**DOI:** 10.1186/s12893-021-01337-6

**Published:** 2021-09-08

**Authors:** Juanjuan Hu, Haiyang Wang, Jianli Chen, Xuelin Pan, Di Deng, Lufang Zhuo, Shixi Liu, Maiyue He, Fei Chen, Hui Yang

**Affiliations:** 1 Department of Otolaryngology, Head and Neck Surgery, West China Hospital, West China Medical School, Sichuan University, No. 37 Guo Xue Xiang, Chengdu, 610041 Sichuan People’s Republic of China; 2 Department of Burn and Plastic Surgery, Dazhou Central Hospital, Dazhou, Sichuan People’s Republic of China; 3 Department of Radiology, West China Hospital, West China Medical School, Sichuan University, Chengdu, Sichuan People’s Republic of China

**Keywords:** Laryngeal paraganglioma, Subglottic paraganglioma, Thyroid paraganglioma, Tracheal paraganglioma, Case report

## Abstract

**Background:**

Subglottic paragangliomas (PGs) are exceptionally rare and unpredictable, occasionally presenting at an atypical location. There are three different clinical forms of subglottic PGs: intraluminal (tracheal PGs), extraluminal (thyroid PGs) and the mixed type (both intraluminal and extraluminal, mixed-subglottic PGs). These tumors are usually misdiagnosed as other relatively common primary thyroid or laryngotracheal tumors, and the treatment is troublesome.

**Case presentation:**

A 22-year-old male patient with subglottic PGs has been successively misdiagnosed as thyroid tumors and subglottic hemangiomas, and lastly underwent local extended lumpectomy and laryngotracheal reconstruction with a pedicled thoracoacromial artery perforator flap (PTAPF). The patient was decannulated successfully after the second-stage tracheal reconstruction with a local flap, and no evidence of local recurrence and distant metastasis of the tumor until now.

**Conclusion:**

Subglottic PGs can be easily misdiagnosed as laryngotracheal or thyroid tumors when presented at an atypical location. It is essential for otolaryngologists and head and neck surgeons to remain vigilant against these tumors. If the tumor is not diagnosed or removed completely, patients may encounter a risk of lethal paroxysm, which is incredibly troublesome.

## Background

Paragangliomas (PGs) are slow-growing, well vascularized and rare-neuroendocrine neoplasms that arise from the paraganglion system. The paraganglia are tissues with common embryological origin that widely distributed throughout the body [1]. PGs have been described in many sites and, therefore, are classified according to their anatomical site of origin and functional activity. In the head and neck regions, PGs are usually biochemically silent and associated with the carotid body, vagus nerve, middle ear or jugulotympanic paraganglia [2]. Additionally, rare cases of head and neck PGs have been reported affecting the larynx or thyroid.

Laryngeal PGs, originating in the superior or inferior laryngeal paraganglion, may be either supraglottic, glottic, or subglottic [3, 4]. More than 90% of laryngeal PGs occur in the supraglottic larynx and originate from the superior laryngeal paraganglia [5]. The superior laryngeal paraganglia are found around the upper anterior third of the ventricular fold near the superior edge of the thyroid cartilage [6, 7]. Subglottic PGs are exceptionally rare and entirely unpredictable [8], originating along the inferior laryngeal paraganglion. The inferior paraganglia are larger and usually situated near the lateral margin of the cricoid cartilage in the cricotracheal membrane along the course of the recurrent laryngeal nerve. Occasionally, it can also be present within the capsule of the thyroid gland. Subglottic PGs are presented with three different clinical forms [9] due to location changes of the inferior paraganglia. The first type is purely intraluminal, often labelled as tracheal PGs, and is located below the true vocal cords. Currently, only 13 tracheal PGs have been reported in the literature [10, 11]. The second type is extraluminal; this type often refers to thyroid PGs appearing as a cervical mass in the thyroid region with just over 60 reports in the literature since its first description by Haegert DG in 1974 [12, 13]. The final clinical form combines the types mentioned above with both intraluminal and extraluminal presentations; these tumors are categorized as the mixed type and are extremely rare. Compared with the first two types, the mixed type of subglottic PGs can be easily misdiagnosed as laryngotracheal or thyroid tumors. This occurs due to their particularly low prevalence, hidden symptoms, occasionally displacement into adjacent structures, and the histopathologic similarities with other more frequently diagnosed tumors [14]. Currently, only five well-documented cases of mixed type PGs are reported in the scientific literature published in the English language (Table 1). All of them were initially thought to be laryngeal invasion with thyroid origin. Here we describe an additional case of the mixed-subglottic PGs with a systematic literature review and provide a feasible management strategy.

**Table 1:** Important aspects of the mixed type of subglottic PGs described in the literatures

Author	Year	Sex-age (Y)	Initial operation		Second operation		
			Pathology	Surgery	Pathology	Resection	Reconstruction
Olofsson [18]	1984	F/44	Hemangiopericytoma	Tracheostomy and biopsy	PG	Laryngectomy, left thyroid lobectomy, upper trachea, partial pharyngectomy	Not mentioned
Brownlee [34]	1992	F/27	MTC	Right thyroid lobectomy	PG	Right half of the cricoid and first tracheal ring	Cartilage rib and auricular cartilage
Kronz [26]	2000	F/52	MTC	Left thyroid lobectomy	PG	Completion thyroidectomy, total laryngectomy, pharyngectomy, esophagectomy, central and bilateral neck dissections	Not mentioned
Hinojar [14]	2003	F/53	MTC	Total thyroidectomy	PG	The right anterolateral half of the cricothyroid membrane, the cricoid, and the first three tracheal rings	Free septal cartilage graft
Michaelson [16]	2005	F/50	MTC	Near total thyroidectomy without neck dissection	PG	Three tracheal rings	End to end

*Y* years old; *F* female; *MTC* medullary thyroid carcinoma; *PG* paraganglioma

## Case presentation

A 22-year-old man presented with a history of recurrent hemoptysis of small volume for a duration of two years. He also experienced progressive dyspnea for three months. The patient had no other complaints, such as hoarseness, dysphagia, or any other symptoms. Following the development of stridor in April 2017, the patient underwent a tracheostomy in the emergency department at West China Hospital, with dyspnea relieved markedly. The physical examination didn’t reveal any special issues except for a scar on the neck. A review of the patient’s medical history revealed that 7 years prior, he had undergone left thyroid lobectomy without the neck dissection for a palpable 3-cm left thyroid mass in a local hospital. Moreover, the post-operative histopathology was unknown.

**Fig. 1:**
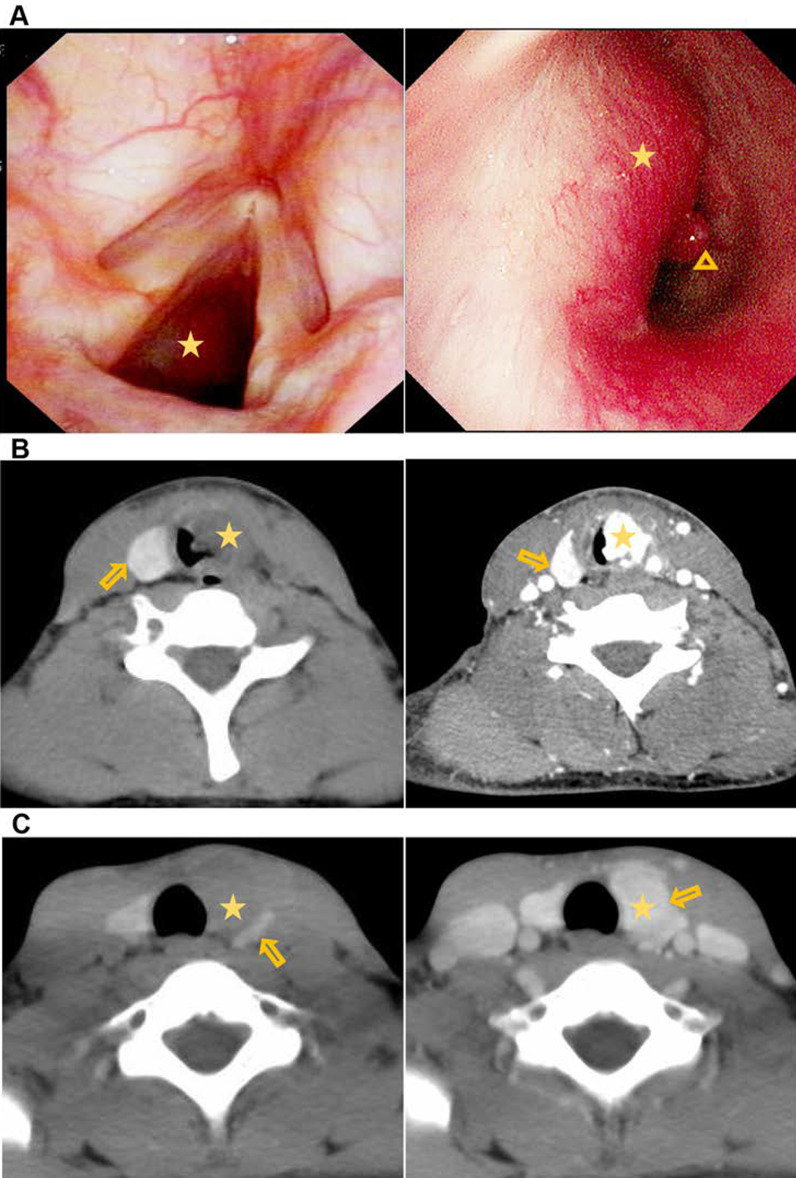
Preoperative imaging of the new reported case. **A** the smooth and sessile vascular tumor (labeled with asterisk) was seen in the subglottis and vascular proliferation in a pseudo angiomatosus (labeled with triangle) was captured on the tumor surface by flexible fiberoptic bronchoscope (the right image is a close view of the left image). **B** CT scans with plain (the left) and contrast-enhanced (the right) image showing the intraluminal and extraluminal involvement of left subglottic larynx (labeled with asterisk). The right lobe of the thyroid (labeled with arrow) was present while the left lobe and isthmus of thyroid were absent. **C** the demarcation line was not clearly recognized between the tumor (labeled with asterisk) and the thyroid (labeled with arrow) by plain CT scan (the left) and contrast-enhanced CT scan (the right) before thyroidectomy (7 years prior to this study)

He was then hospitalized at the respiratory department after tracheostomy. Flexible fiberoptic bronchoscope revealed a smooth and sessile vascularized mass, located 0.5–1.5 cm below the glottis, causing lumen narrowing by approximately 80% (Fig. 1a). Pathological analysis of a biopsy from the mass described it as lobulated capillary hemangioma. Furthermore, enhanced CT scans confirmed a bulky and vascularized tumor located primarily in the cervical trachea, which may have originated from the left tracheoesophageal groove with intraluminal exophytic and extratracheal components (Fig. 1b). Upon reviewing CT scans before the thyroidectomy 7 years prior, images showed that the tumor was also hyper-vascularized, though limited to the thyroid region with ambiguous tracheoesophageal sulcus (Fig. 1c). Lab work, which included complete blood cell count, C-reactive protein, calcitonin and thyroid function (TSH, FT3 and FT4), were within normal limits, which help exclude from acute infection, medullary thyroid carcinoma (MTC) and thyroid dysfunction.

**Fig. 2:**
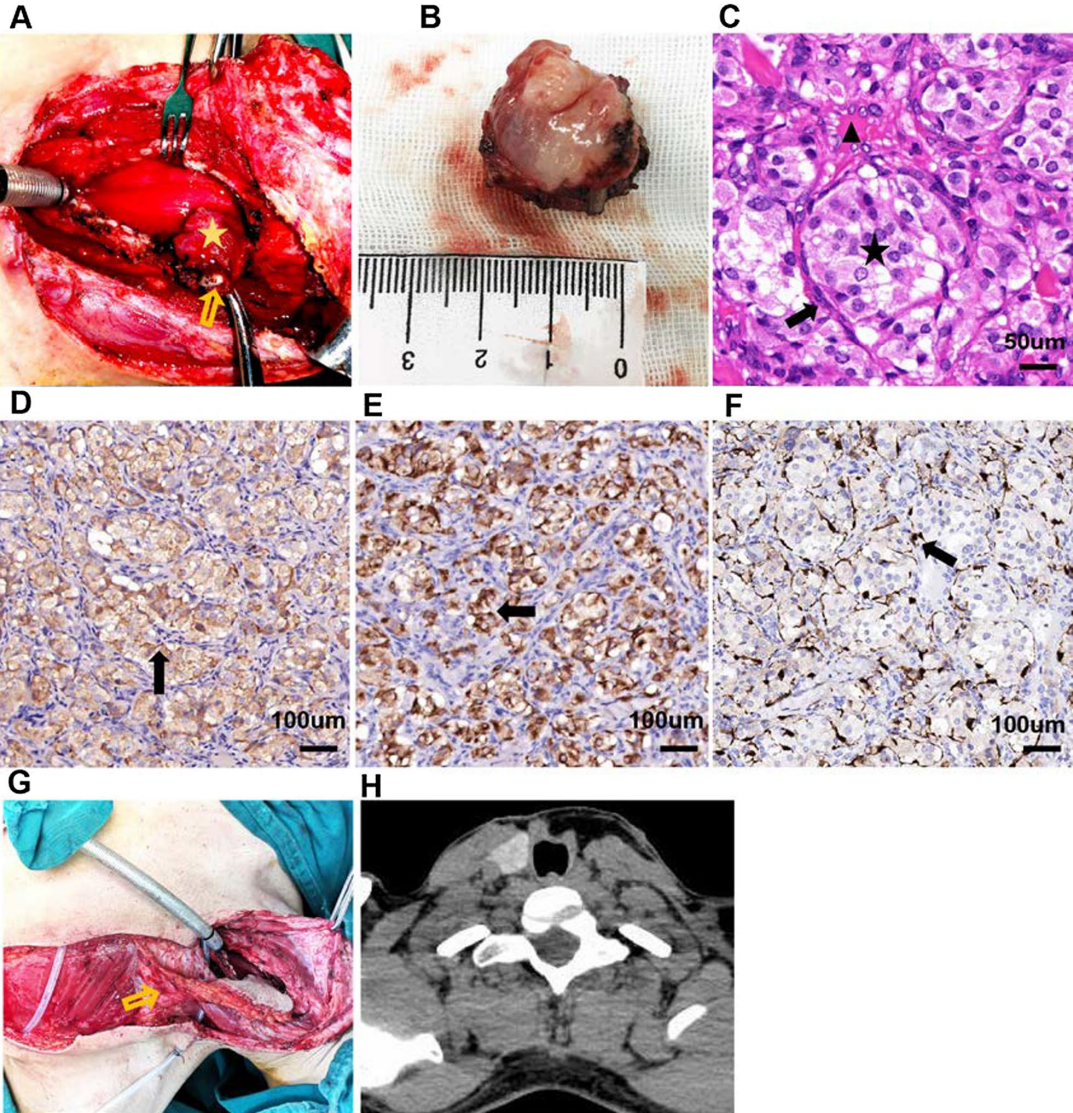
Operative imaging and pathological analysis of the new reported case. **A** intra-operative view of the tumor (labeled with asterisk) and the cricoid cartilage (labeled with arrow). **B** the gross specimen consisted of a 3.0- × 3.0- × 2.0-cm irregular, tan–pink mucosal lesion attached to the lower portion of the cricoid cartilage and the first three tracheal cartilages. **C** the chief cells (labeled with asterisk) show abundant and pale pink cytoplasm, and round nuclei with nucleoli. The peripheral sustentacular cells (labeled with arrow) are small and long with oval nuclei. Vascular sinusoids (labeled with triangle) are around the organoid pattern of “zellballen” (Hematoxylin–Eosin × 40), scale bar: 50 μm. **D**, **E** immunostaining analysis shows that chief cells are positive for chromogranin (**D**) and synaptophysin (**E**) in the cytoplasm (arrow, original magnification ×20), scale bar: 100 μm. **F** the peripheral sustentacular cells are outlined by S100 immunostaining (arrow, original magnification ×20). **G** intra-operative view of reconstruction of the defect with PTAPF (labeled with arrow), scale bar: 100 μm. **H** CT scans shows the reconstructed trachea with successful decannulation and no sign of tumor recurrence at the third year post-operatively

With the initial consideration of subglottic or cervical tracheal hemangioma based on the biopsy results, the patient was referred to the department of otolaryngology for sclerotherapy combined with CO_2_ laser using suspension laryngoscope under general anaesthesia. The transoral surgery was aborted due to the potential presence of an undefined solid mass and possible excessive bleeding. Then, intra-operative frozen biopsy was performed and subsequently indicated a vascularized tumor characterized by small vessels and atypical proliferative cells. Therefore, a lateral cervical approach was taken to remove the tumor. The tumor was located in the subglottis with involvement of the lower portion of the cricoid cartilage and bulged into the left perichondrium of the first three tracheal cartilages (Fig. 2a). The mass was complete en-bloc resected, including the left lateral part of the cricoid cartilage and the first six tracheal rings with negative margins on permanent pathology (Fig. 2b). Histologically, hematoxylin and eosin staining showed a characteristic “zellballen” pattern growth traversed by a delicate capillary network (Fig. 2c). Immunohistochemical studies determined the followings: The chief cells were positive for chromogranin A (abcam, ab52983, 1:250) (Fig. 2d) and synaptophysin (abcam, ab8049, 1:200) (Fig. 2e), and the sustentacular cells were stained positively for S-100 (abcam, ab136629, 1:200) (Fig. 2f). In addition, the tumor was negative for calcitonin, keratin, carcinoembryonic antigen (CEA), and thyroid transcription factor 1 (TTF-1). Finally, the tumor was diagnosed as paraganglioma. The defect, created by complete removal of tumors with six tracheal rings and partial cricoid cartilage resection, was challenging to reconstruct by end-to-end anastomosis. Simultaneously, a pedicled thoracoacromial artery perforator flap (PTAPF) was harvested to reconstruct the left lateral wall of the trachea and cricoid cartilage. The anterior wall of the defect was left to second-stage reconstruction (Fig. 2g).

The patient was uneventful post-operatively and discharged with tracheostomy cannula. Six months post-operatively, the second-stage tracheal reconstruction was conducted with a local flap and the patient decannulated successfully. In addition, no evidence of local recurrence or distant metastasis was noted during the 3-year follow-up (Fig. 2h).

## Discussion and conclusions

**Fig. 3:**
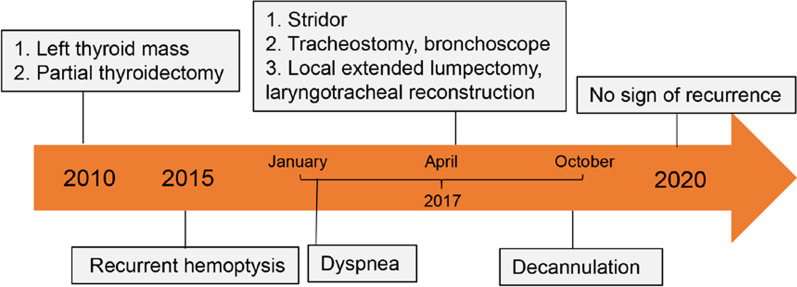
The timeline of medical record of the new reported case

This young man has been experienced two misdiagnoses successively and tortuous medical treatment processes (Fig. 3), two questions remained to be addressed. The first was whether there was any relationship between the initial thyroid mass detected 7 years prior and the subsequent cervical-tracheal tumor detected in April 2017. The second was why the pathological interpretation of the biopsy obtained by fiberoptic bronchoscopy suggests lobular capillary hemangioma, while the final pathology was established as PG. Accordingly, we analyzed the clinical presentations and histopathological features, reviewed relevant literature, and highlighted diagnostic pitfalls and subsequent management problems that may occur with subglottic PGs.

### Pitfalls in clinical diagnosis of subglottic PGs at atypical location

Epidemiologically, subglottic PGs typically originate between the fourth and the sixth decade of life with a female preponderance and a right side predominance [3]. While the new case described here did not accord with this general pattern regarding age, sex and laterality.

Clinical characteristics associated with subglottic PGs are determined by the sizes and locations of the tumors (Table 2). For lesions in the trachea, tumors may possess an unimpeded growth, resulting in developing into the presentations of dysphagia, pain, cough or hemoptysis. These tumors often reach a large size or grow circumferentially before the onset of airway obstruction, needing emergent intubation or tracheostomy. For lesions in the thyroid region, most patients have asymptomatic thyroid nodule for several years. These tumors are detected incidentally by radiographic imaging or ultrasonography. On the other hand, a few symptomatic patients with thyroid PGs can present dysphagia, dyspnea, stridor, or hemoptysis, which are easily confused with advanced thyroid malignancies due to intraluminal involvement of the respiratory-digestive tract. These tumors are categorized as the mixed type and are extremely rare.

**Table 2:** Clinical characteristics of three subglottic PGs subtypes

Tumors	Tracheal PGs	Thyroid PGs	Mixed type
Symptoms	Dysphagia, pain, cough, hemoptysis or stridor	Asymptomatic	Dysphagia, pain, cough, dyspnea, hemoptysis, or stridor
Signs	Red and smooth subglottic mass	Neck mass	Subglottic and neck mass
Location	Intraluminal	Extraluminal	Both intraluminal and extraluminal
Treatment	Tracheostomy and laryngotracheal reconstruction	Thyroidectomy	Thyroidectomy, tracheostomy and laryngotracheal reconstruction

*PG* paraganglioma

As previously noted, only five cases of mixed type have been reported in the literature, and all the patients were females with presumed thyroid mass initially (Table 1). The new case reported in this study was primarily presented as an asymptomatic thyroid mass that grew inwards with laryngotracheal invasion. Thus, symptoms or signs of subglottic PGs provided limited diagnostic information due to characterless presentation, variable duration (range from several days to years), and particularly constant location related to trachea, thyroid, or both of them [15, 16].

To our knowledge, there is currently no recognized consensus for diagnostic imaging and investigations. Concerning the initial presentation, preoperative CT/ MRI scanning provides the performance of a non-invasive assessment of tumor size, location and vascularity, and the possibility of ruling out the existence of other associated lesions [17]. Subglottic PGs' vascular nature is characterised by a dominant feeder vessel, including the thyrocervical trunk or the superior/inferior thyroid artery [18]. Other tumors in the subglottis or the thyroid region, on the other hand, often have a variable blood supply [19]. CT angiography presents the vascular relationships with a profuse and homogeneous tumor blush in the capillary phase or a major nutrient vessel [20], and MRI portrays the classic “salt and pepper” appearance. Anatomic and functional imaging are keys to the diagnosis of clinically suspected PGs and the development of individualized treatment strategy [21].

### Pitfalls in pathologic diagnosis of subglottic PGs at atypical location

The fine needle aspiration or biopsy is highly sensitive and specific. Strikingly, if tumors are characteristically hypervascular, biopsy should be averted or conducted strictly with airway management as there is a risk of uncontrollable haemorrhage. Accordingly, few subglottic PGs have been diagnosed before the operation and were often misinterpreted as other relatively common primary thyroid or laryngotracheal tumors. This was mainly due to cytologic and histopathologic complexities and similarities [14, 22–24].

Morphologically, tracheal PGs are red, subepithelial, and highly vascular lesions without ulceration of the overlying mucosa. Such PGs are easily misdiagnosed as hemangiomas when there are areas of haemorrhage and dense fibrous capsules with streaks of fibrous septal [4, 18]. For the new case reported here, vascular proliferation is present in the mucosa adjacent to the tumor (Fig. 2a). Moreover, the initial biopsy with fiberoptic bronchoscope may not have been deep enough, leading to the misdiagnosis of lobulated capillary hemangioma.

Grossly, subglottic PGs in the thyroid region resemble MTC, both exhibit a lobular or nesting growth pattern [25]. Approximately 30% of thyroid PGs reported in the literature were initially misdiagnosed as MTCs that were later overturned by another pathologist [26]. Moreover, four mixed type cases reported in the literature were once diagnosed as MTC (Table 1).

Microscopically, PGs are composed of two cell types: the centrally located chief cells and the peripherally located sustentacular cells. These cells form loose nests called “zellballen” that are surrounded by an extensive network of vascular sinusoids, which could be confused with follicular formations in follicular thyroid carcinoma (FTC) [27].

Therefore, an accurate diagnosis needs to combine light microscopy and immunohistochemical staining (Table 3). The chief cells of PGs are typically positive for neuroendocrine markers, including neuro-specific enolase, chromogranin A, and synaptophysin, and negative for epithelial markers such as cytokeratin, calcitonin, epithelial membrane antigen (EMA) and carcinoembryonic antigen (CEA). The sustentacular cells are almost always positive for S-100 protein [16]. The presence of neuroendocrine markers helps differentiate PGs from other tumors originating from the neural crest, whereas the absence of the epithelial and calcitonin staining differentiate the PGs from the laryngeal carcinoids and small cell carcinomas [12]. Negative staining for keratins, cytokeratins, thyroid transcription factor 1 (TTF-1), carcinoembryonic antigen (CEA) and calcitonin excludes primary thyroid neoplasms, including MTC and FTC, which are positively stained with CEA and calcitonin markers [12]. Chromogranin A can be positive in some cases of FTC, causing diagnostic confusion. However, these tumors show strong membranous staining for molecular immunology Borstel number 1 (MIB-1), which is negative in PGs and any other thyroid neoplasm [27].

**Table 3:** Differentiating neuroendocrine tumors with immunohistochemical markers

Immunohistochemical markers	Paraganglioma	Typical carcinoid	Atypical carcinoid	Small cell neuroendocrine tumor	MTC	FTC
1.Neuro-specific enolase	(+)	(+)	(+)	(+)	(+)	(−)
2.Chromogranin A	(+)	(+)	(+)	(+)	(+)	(+)
3.Synaptophysin	(+)	(+)	(+)	(+)	(+)	(−)
4.Glial fibrillary acidic protein*	(+)	(−)	(−)	(−)	(+)	(−)
5.S-100 protein*	(+)	(−)	(−)	(−)	(−)	(−)
6.Cytokeratin	(−)	(+)	(+)	(+)	(+)	(+)
7.Calcitonin	(−)	(+)	(+)	(+)/(−)	(+)	(+)
8.CEA	(−)	(+)	(+)	(+)	(+)	(+)
9.EMA	(−)	(+)	(+)	(+)	(+)	(+)
10. TTF1	(−)	(−)	(−)	(+)/(−)	(+)	(+)
11. MIB-1	(−)	(−)	(−)	(−)	(−)	(+)

*MTC* medullary thyroid carcinoma; *FTC* follicular thyroid carcinoma; *CEA* carcinoembryonic antigen; *EMA* epithelial membrane antigen; *TTF-1* thyroid transcription factor 1; *MIB-1* molecular immunology Borstel number 1

*Positivity only in the sustentacular cells

### Individualized treatment and the prognosis of subglottic PGs

Management option for PGs is accomplished by surgery, radiotherapy, or observation [28]. The choice depends on several factors, including the location, tumor size, extent of the tumor, patient age and health, the anticipated morbidity of the treatment alternatives, and preferences of the patient and physician. It has been proved that radiotherapy for PGs was safe and efficient in head and neck region, with successful local and distant control of more than 95% during 15 years’ follow-up [29]. Considering the cytology and frozen-section histology of subglottic PGs are generally non-diagnostic or at least uncertain [30], complete surgical resection is still the optimal choice for subglottic PGs. An open procedure is preferred as endoscopic excisions have a high recurrence rate of 80%, in addition to the risk of uncontrollable haemorrhage [16]. Usually, prophylactic neck dissection is not necessary when there is no evidence of lymphatic metastasis. For the thyroid PGs of small masses without adjacent invasion, a thyroid lobectomy can be performed. Laryngectomy is rarely indicated for both tracheal PGs and thyroid PGs due to organ-preservation strategy. Nevertheless, the mixed type of subglottic PGs poses different problems due to their relationship with both the airway and thyroid. First, more extensive surgery, including the surrounding mucosa and adjacent perichondrium, is imperative to reduce recurrence [31]. Second, when surgical resection of subglottic PGs is performed with wedge-or sleeve-resection of the invaded cricoid cartilage and cervical trachea, laryngotracheal reconstruction poses a great challenge for head and neck surgeons. The PTAPF, which is of strong vasculature, consistent pedicle length and caliber size and limited donor site morbidity, has been previously described to close complex hypopharyngeal and laryngeal defects [32]. In this case, the size of defect and demand of functional preservation were all in favour of this pedicled flap. Last, for the tracheal PGs and mixed type, temporary tracheostomy is often performed in order to open the airway pre-operatively at the acute onset of dyspnea. This helps complete resection and simultaneous reconstruction intraoperatively. Additionally, in the post-operative period, temporary tracheostomy helps uncomplicated recovery with resolution of post-operative edema [33].

An accurate survival rate for subglottic PGs is hard to obtain due to their rarity and diagnostic uncertainty. Therefore, long-term clinical follow-up is required to check for recurrence and distant metastases. Recurrence occurs mainly in the mixed type of subglottic PGs and cases with endoscopic surgery [34]. Until now, no cases of subglottic PGs with distant metastases have been reported.

In conclusion, subglottic PGs are rare, and most otolaryngologists and head and neck surgeons may never encounter them. Patients with subglottic PGs may show up at various medical and surgical specialists in different departments or hospitals. Therefore, it is crucial for otolaryngologists and head and neck surgeons to remain vigilant against these tumors. Presently, surgery remains the mainstay of treatment and offers the best results to these patients. If the tumor is not diagnosed or resected completely, patients may encounter a risk of lethal paroxysm, which is incredibly troublesome.

## Data Availability

All data generated or analysed during this study are included in this published article.

## Abbreviations

PGs: Paragangliomas
CEA: Carcinoembryonic antigen
TTF-1: Thyroid transcription factor 1
PTAPF: Pedicled thoracoacromial artery perforator flap
MTC: Medullary thyroid cancer
FTC: Follicular thyroid carcinoma
Y: Years old
F: Female
EMA: Epithelial membrane antigen
MIB-1: Molecular immunology Borstel number 1
